# A Case Report of Wet Beriberi Due to Excessive White Rice Consumption in an Elderly Male Patient: A Potentially Forgotten and Underrecognized Disease

**DOI:** 10.7759/cureus.67445

**Published:** 2024-08-21

**Authors:** Satoshi Kurisu, Hitoshi Fujiwara

**Affiliations:** 1 Department of Cardiology, National Hospital Organization Hiroshima-Nishi Medical Center, Otake, JPN

**Keywords:** case report, cardiac output, echocardiography, diuretics, thiamine

## Abstract

Thiamine deficiency can cause various clinical manifestations. Wet beriberi, a phenotype of thiamine deficiency, is often underdiagnosed in clinical practice due to the nonspecificity of symptoms. An 83-year-old man presented to a primary care clinic with a two-month history of progressing edema in the scrotum and lower extremities. The patient reported a weight gain of 10 kg and was treated with diuretics. However, his condition did not improve even after four weeks. The patient was referred to our hospital for further cardiac evaluation. Transthoracic echocardiographic findings were suggestive of a high cardiac output (CO) state, and the thiamine level was decreased. Further medical interview revealed that the patient had a habit of eating two or three large bowls of white rice with a few side dishes for breakfast and dinner. The hemodynamic evaluation revealed high CO and low systemic vascular resistance. The patient's weight decreased from 56.6 to 52.4 kg in the first 2 days after thiamine administration. Six days later, his weight further decreased to 50.8 kg and edema disappeared completely. Clinicians should be aware that excessive consumption of white rice with few side dishes may lead to thiamine deficiency. This case highlights the importance of considering wet beriberi as a cause of excessive edema with high CO state.

## Introduction

Thiamine (vitamin B1) is a water-soluble vitamin available in grains, fruits, vegetables, and meats, and it is an essential coenzyme for oxidative cellular metabolism. Thiamine deficiency, owing to an imbalanced diet or chronic alcoholism, can cause various clinical manifestations such as anorexia, fatigue, and cardiovascular and neurological disturbances [1-4]. There are two major phenotypes of thiamine deficiency: wet beriberi and dry beriberi. The former affects the cardiovascular system, whereas the latter affects the nervous system. Wet beriberi is typically characterized by low systemic vascular resistance (SVR) with a compensatory high cardiac output (CO) state [1-6]. The reduction in SVR decreases arterial volume and renal hypoperfusion, causing neurohumoral activation and plasma volume expansion [6]. As a result, patients with wet beriberi commonly present with edema. Unfortunately, wet beriberi is often underdiagnosed in clinical practice due to the nonspecificity of symptoms.

Herein, we report a case of wet beriberi in an elderly man, which was induced by excessive consumption of white rice and was resolved completely with appropriate thiamine administration.

## Case presentation

An 83-year-old man with hypertension, who had been treated with telmisartan (40 mg), presented to a primary care clinic with a two-month history of progressing edema in the scrotum and lower extremities. The patient reported a weight gain of 10 kg and was treated with azosemide (30 mg/day) and spironolactone (25 mg/day). However, his condition did not improve even after four weeks. The patient was referred to our hospital for further cardiac evaluation.

**Figure 1:**
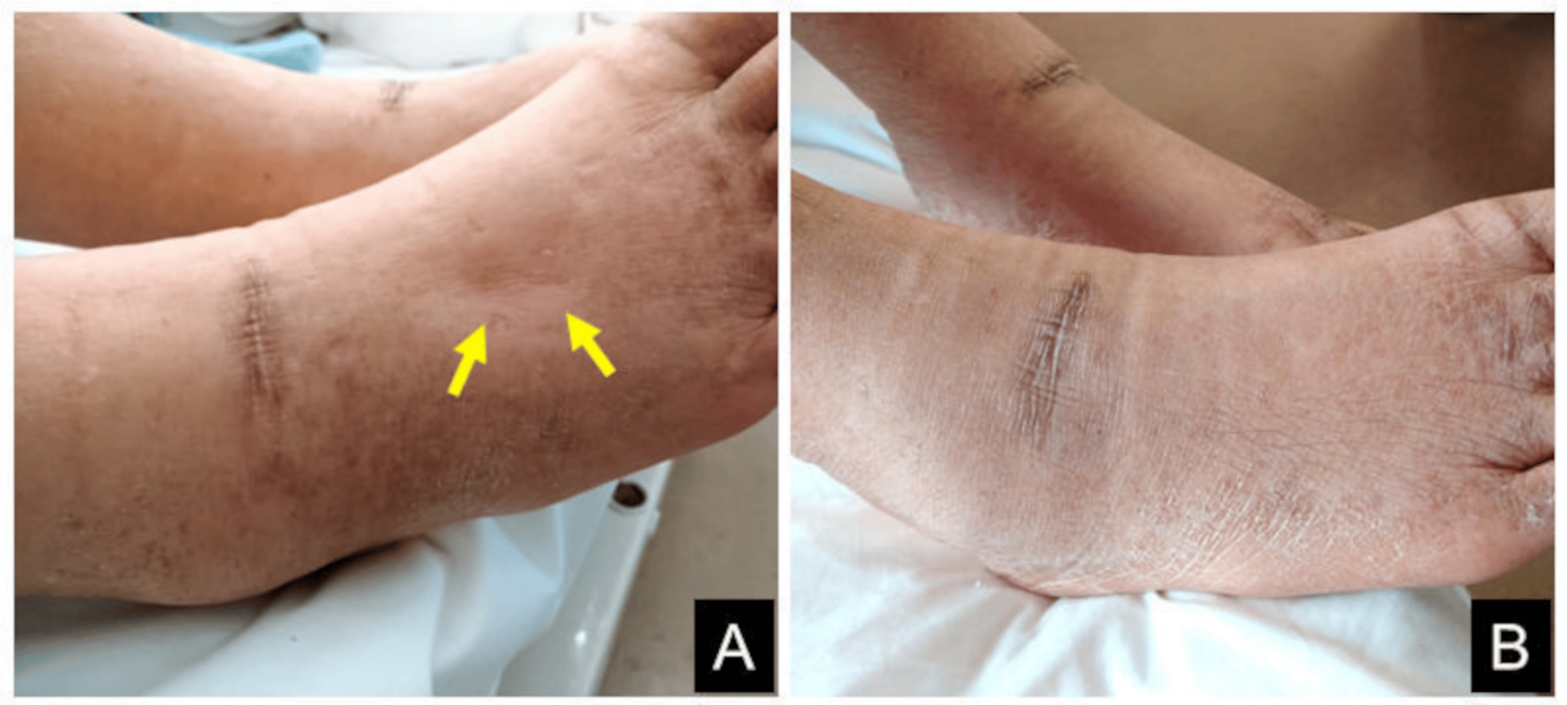
Right foot before and after thiamine administration. There was excessive pitting edema (A, yellow arrows), which disappeared completely six days after thiamine administration (B)

The patient had neither a habit of alcohol consumption nor any history of surgical operations. On physical examination, his pulse rate was 80 bpm, blood pressure 144/72 mmHg, body weight 57 kg, and body mass index 23.7 kg/m^2^. The patient had a good appetite, and his body build was normal. Excessive pitting edema in the lower extremities (Figure 1A, yellow arrows) was found. He had no neurological symptoms.

N-terminal pro-brain natriuretic peptide (NT-proBNP) level was 529 pg/mL (normal: <126 pg/mL) (Table 1). Liver, renal, and thyroid functions were almost normal.

**Table 1:** Laboratory data before and after thiamine administration. At the initial presentation, the NT-proBNP level was 529 pg/mL. Liver, renal, and thyroid functions were almost normal. Six days after thiamine administration, NT-proBNP and thiamine levels returned to normal values IU: international units; NT-proBNP: N-terminal pro-brain natriuretic peptide

Variable	Initial presentation	Six days after thiamine	Reference range
White blood cell counts	5.0 × 10^3^ cells/mm^3^	5.4 × 10^3^ cells/mm^3^	3.3-8.6 × 10^3^ cells/mm^3^
Red blood cell counts	2.93 × 10^6^ cells/mm^3^	3.35 × 10^6^ cells/mm^3^	4.35-5.55 × 10^6^ cells/mm^3^
Hemoglobin	9.8 g/dL	12.3 g/dL	13.7-16.8 g/dL
Platelet counts	178 × 10^3^ cells/mm^3^	232 × 10^3^ cells/mm^3^	158-348 × 10^3^ cells/mm^3^
Aspartate aminotransferase	37 U/L	23 U/L	13-30 U/L
Alanine aminotransferase	31 U/L	20 U/L	10-42 U/L
Lactate dehydrogenase	278 U/L	197 U/L	124-222 U/L
Creatine kinase	218 U/L	-	59-248 U/L
Total protein	6.3 g/dL	6.6 g/dL	6.6-8.1 g/dL
Albumin	3.7 g/dL	3.7 g/dL	4.1-5.1 g/dL
Blood urea nitrogen	28.4 mg/dL	26.6 mg/dL	8-20 mg/dL
Creatinine	0.87 mg/dL	1.03 mg/dL	0.65-1.07 mg/dL
C-reactive protein	0.11 mg/dL	-	0-0.14 mg/dL
NT-proBNP	529 pg/mL	97 pg/mL	<126 pg/mL
Thyroid-stimulating hormone	4.98 μIU/mL	-	0.61-4.23 μIU/mL
Free triiodothyronine	2.86 pg/mL	-	1.68-3.67 pg/mL
Free thyroxine	1.01 ng/dL	-	0.7-1.48 ng/dL
Thiamine (vitamin B1)	23 ng/mL	81 ng/mL	24-66 ng/mL
pH	7.394	7.399	7.35-7.45
HCO^3-^	27.3 mEq/L	23.1 mEq/L	22-26 mEq/L
Lactate	0.9 mmol/L	0.8 mmol/L	0.5-1.5 mmol/L

**Figure 2:**
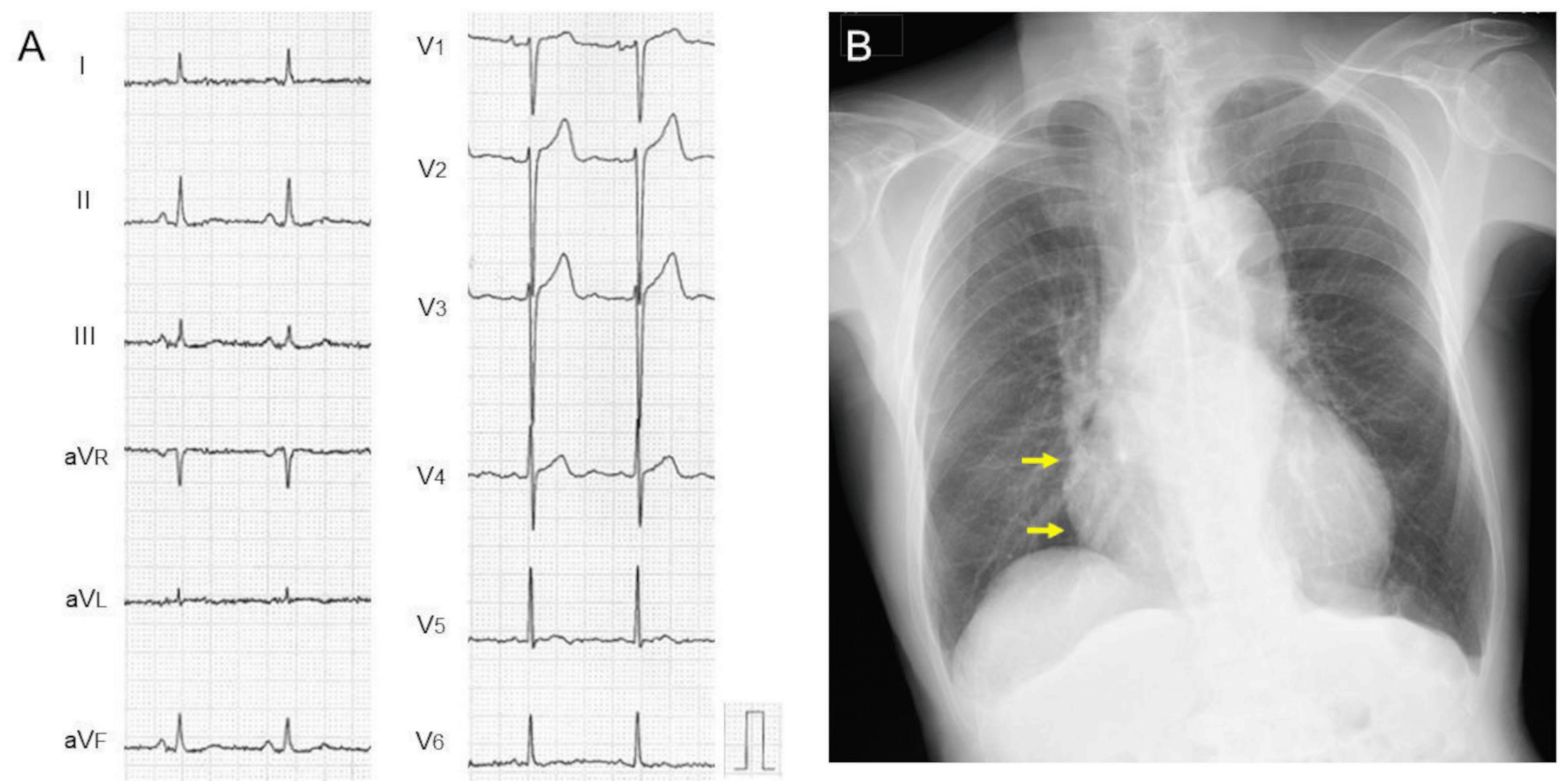
(A) Electrocardiogram showing no significant abnormalities except for flat T-waves in leads V5,6. (B) Chest radiograph showing an enlarged cardiac silhouette, especially the right atrium, with a cardiothoracic ratio of 58% (yellow arrows)

An electrocardiogram showed no significant abnormalities except for flat T-waves in leads V_5,6_ (Figure 2A). A chest radiograph showed an enlarged cardiac silhouette, especially the right atrium, with a cardiothoracic ratio of 58% (Figure 2B, yellow arrows).

**Figure 3:**
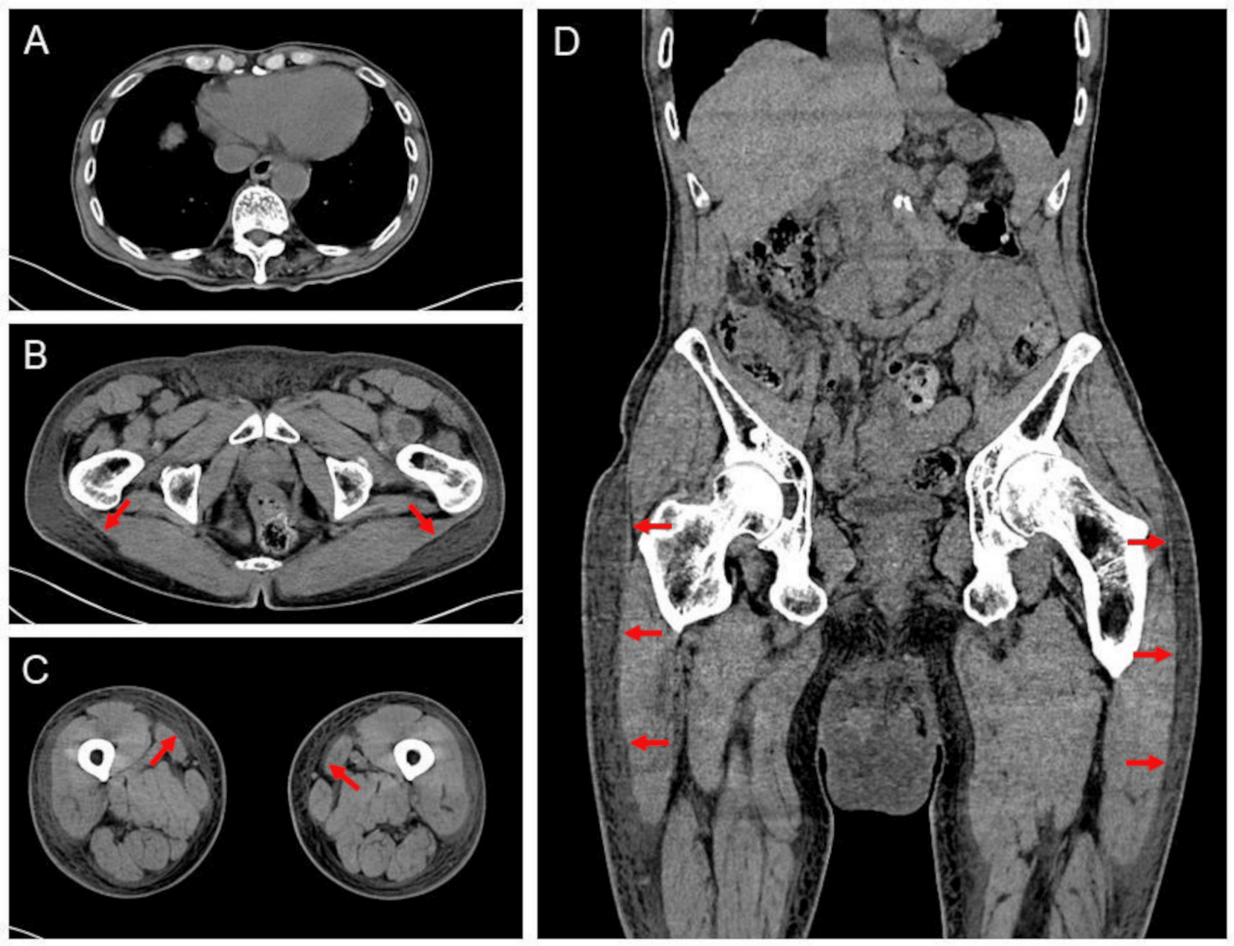
Computed tomographic images revealed no pleural effusion in the thorax (A). There were no significant abnormalities, such as malignancies inhibiting venous return in the abdomen. Excessive edema was distributed below the waist (B-D, red arrows)

Computed tomographic images revealed no pleural effusion in the thorax (Figure 3A). There were no significant abnormalities, such as malignancies inhibiting venous return in the abdomen. It was noted that excessive edema was distributed below the waist (Figures 3B-3D, red arrows).

**Figure 4:**
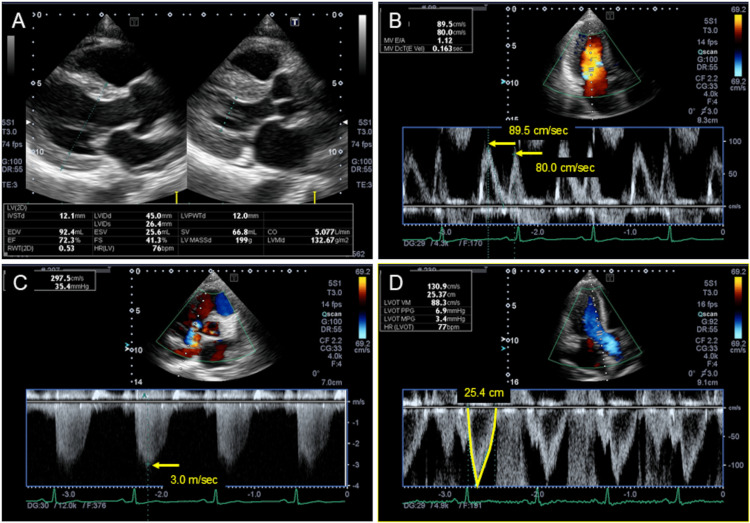
(A) A transthoracic echocardiogram revealed LV hyperkinesia with an ejection fraction of 72%. Doppler echocardiographic measurements disclosed the following values: (B) early-to-late mitral peak flow velocity ratio, 1.1; (C) peak tricuspid regurgitation velocity, 3.0 m/second; and (D) LV outflow tract velocity time integral, 25.4 cm LV: left ventricular

A transthoracic echocardiogram (TTE) revealed left ventricular (LV) hyperkinesia with an ejection fraction of 72% (Figure 4A). Doppler echocardiographic measurements disclosed the following values: early-to-late mitral peak flow velocity ratio of 1:1 (Figure 4B), peak tricuspid regurgitation velocity of 3.0 m/second (Figure 4C), and LV outflow tract velocity time integral of 25.4 cm (Figure 4D). These TTE findings suggested a high CO state. Accordingly, serum thiamine was additionally measured, and right-sided cardiac catheterization was planned.

**Figure 5:**
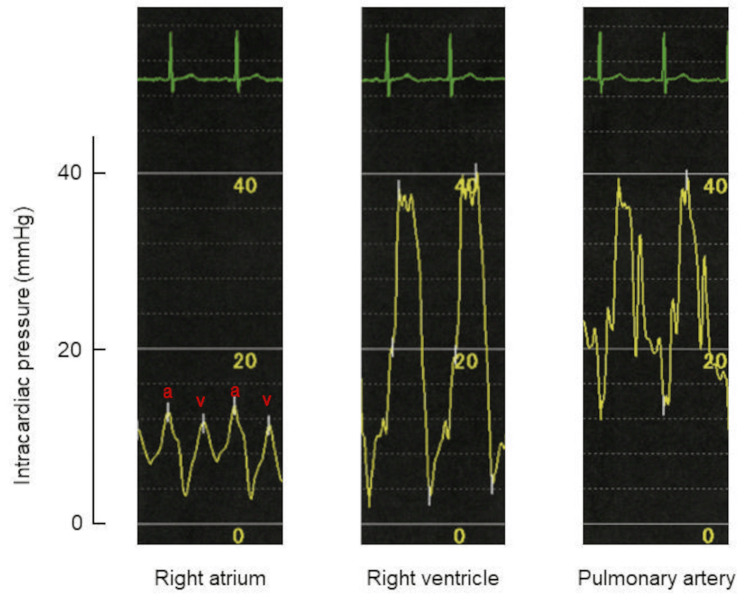
Intracardiac pressure measurements. Hemodynamic evaluation using a Swan-Ganz catheter revealed increased intracardiac pressures in the right atrium, right ventricle, and pulmonary artery a: a-waves; v: v-waves

One week later, the patient was admitted for further cardiac evaluation. Before cardiac catheterization, serum thiamine level was noted to have decreased to 23 ng/mL (normal: 24-66 ng/mL). Further medical interview revealed that the patient had a habit of eating two or three large bowls of white rice with a few side dishes for breakfast and dinner. Hemodynamic evaluation using a Swan-Ganz catheter revealed high CO and low SVR, CO of 8.27 L/minute, SVR of 813 dyne/second/cm^-5^, mean pulmonary capillary wedge pressure of 15 mmHg, and mean right atrial pressure of 9 mmHg (Figure 5).

**Figure 6:**
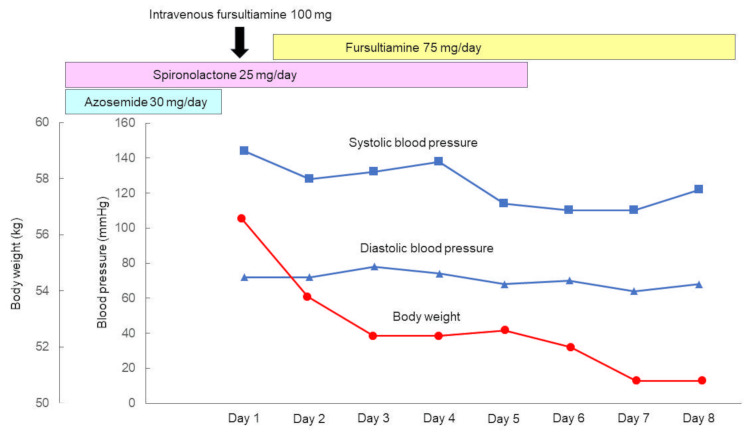
Clinical course during hospitalization. The patient's weight decreased from 56.6 to 52.4 kg in the first two days after thiamine administration

A diagnostic and therapeutic trial of intravenous thiamine (fursultiamine 100 mg) was administered during cardiac catheterization. SVR increased from 813 to 873 dyne/second/cm^-5^ 30 minutes after the administration. The patient was subsequently treated with oral thiamine (fursultiamine 75 mg/day). As shown in Figure 6, the patient's weight decreased from 56.6 to 52.4 kg in the first two days. Six days after thiamine administration, his weight further decreased to 50.8 kg, and edema in his lower extremities disappeared completely (Figure 1B).

NT-proBNP and thiamine levels returned to normal values (Table 1). Based on the remarkable effects of thiamine administration, a definite diagnosis of wet beriberi was made. The patient was discharged after dietary guidance. Oral thiamine was continued for three weeks and then discontinued. During the three-month follow-up, the patient remained in good condition without recurrence of edema.

## Discussion

In this report, we presented a case of wet beriberi in an elderly man with a monotonous diet of white rice as his staple food. We made an early diagnosis of wet beriberi, and his condition improved dramatically after appropriate thiamine administration.

Thiamine is a water-soluble vitamin that serves as a coenzyme in macronutrient oxidation and cellular adenosine triphosphate production. Thiamine deficiency leads to impaired adenosine triphosphate production and adenosine accumulation [7]. It is suggested that released adenosine causes vasodilatation, leading to low SVR with compensatory high CO state and plasma volume expansion [8]. Wet beriberi can be easily overlooked in clinical practice because of the nonspecificity of symptoms [9]. According to the literature review by Lei et al. [10], typical features such as high CO and low SVR are often absent in cases of wet beriberi. This makes the diagnosis of wet beriberi even more difficult. A more severe form of heart failure presenting with low CO and severe lactic acidosis is called Shoshin beriberi [9].

Previous studies have revealed that the major causes of thiamine deficiency in Japan are an imbalanced diet, chronic alcoholism, and a history of gastrectomy [11,12]. At the first medical interview, we ruled out the latter two conditions but failed to find an imbalanced diet because of his normal body build. The high CO state detected by TTE was the first clue to a diagnosis of wet beriberi. There is a wide spectrum of congenital, acquired, and iatrogenic conditions causing high CO state [5,6,13]. In the present case, the patient did not have severe anemia (hemoglobin <8 g/dL), myeloproliferative hematologic disorders, hyperthyroidism, arteriovenous fistula, obesity (body mass index >35 kg/m^2^), chronic obstructive pulmonary disease, or liver disease. This reminded us of the possibility of thiamine deficiency. A decrease in serum thiamine level and the rapid improvement of his condition after thiamine administration led to a final diagnosis of wet beriberi.

A detailed medical interview plays an important role in diagnosing wet beriberi. In the present case, excessive consumption of white rice was identified as the cause of thiamine deficiency. Rice-related beriberi emerged from the polishing of brown to white rice to improve the digestion and taste of cooked rice [14]. This procedure removes the rice germ containing most of the natural thiamine. In Japan, the number of deaths from beriberi peaked around 1923 when the Japanese diet was heavily dependent on rice, exceeding 25,000 [15]. Nutritional interventions have dramatically reduced the subsequent incidence of beriberi. Currently, most clinicians may be less familiar with rice-related beriberi. However, this condition can still occur. Clinicians should be aware that a nutritionally imbalanced diet of excessive white rice and a few side dishes may lead to thiamine deficiency. Once diagnosed, dietary guidance is necessary to prevent its recurrence.

The therapeutic effect of diuretics is mainly mediated by increased urinary salt and volume extraction, while diuretics also increase urinary thiamine extraction depending on the urinary flow rate [16]. The prevalence of thiamine deficiency in patients on diuretics with heart failure has been estimated as high as 30% [17]. Even in the present case, there is a possibility that the use of diuretics may have contributed to thiamine deficiency. Although there are many patients at risk of wet beriberi, it is often not considered in differential diagnoses and, hence, an underdiagnosed condition. Wet beriberi is an easily treatable condition with appropriate thiamine administration. It is important to consider the possibility of wet beriberi in excessive edema with a high CO state. This will lead to an early and accurate diagnosis of wet beriberi.

## Conclusions

In conclusion, we encountered an elderly man with wet beriberi due to excessive consumption of white rice, whose condition improved dramatically after appropriate thiamine administration. Rice-related beriberi emerged from polishing of brown to white rice in order to improve the digestion and taste of cooked rice. This procedure removes the rice germ containing most of the natural thiamine. Currently, most clinicians may be less familiar with rice-related beriberi. However, this condition can still occur. Clinicians should be aware that excessive consumption of white rice with few side dishes may lead to thiamine deficiency. This case highlights the importance of considering wet beriberi as a cause of excessive edema with high CO state.
